# Supplementary material for: Fitness of mCherry Reporter Tick-Borne Encephalitis Virus in Tick Experimental Models
Source: Viruses. 2022 Nov 29;14(12):2673. doi: 10.3390/v14122673 (PMC9781894; doi:10.3390/v14122673)
Supplement: Supplementary file 1 [file viruses-14-02673-s001.zip › viruses-1991962-supplementary.pdf]

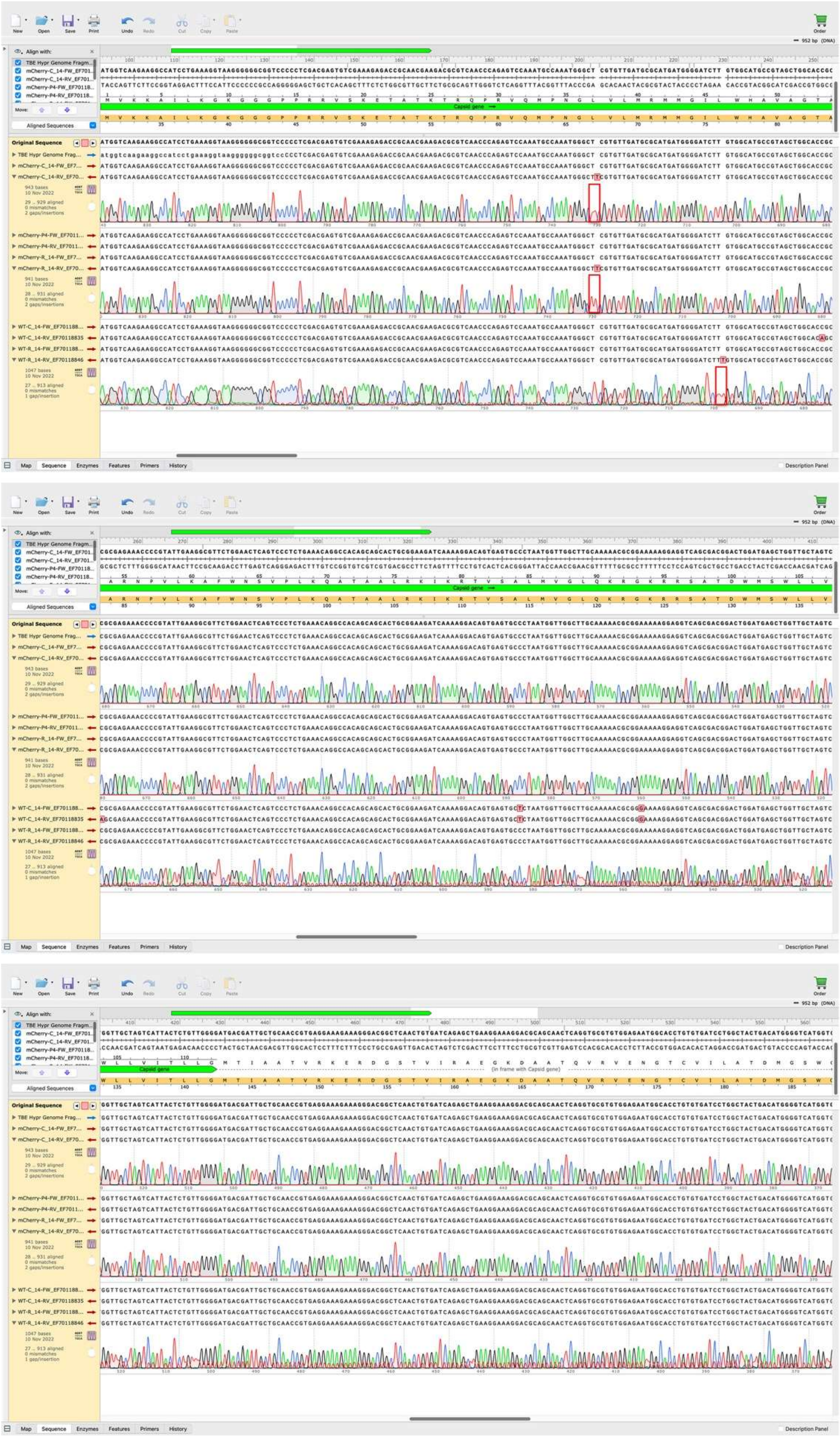

Figure S1. DNA sequencing of capsid gene from mCherry Hypr revertant and WT Hypr variants in tick samples as well as mCherry Hypr passage 4 (revertant). Red rectangles denote occasions of a single nucleotide insertion in reverse reads (demonstrated by the chromatograms) of mCherry Hypr-inoculated as well as WT Hypr-inoculated ticks, apparently caused by the lower quality of the reads. The majority of the reads (7/10) do not contain

a nucleotide insertion, thus confirming a sequence identity of the capsid gene among the viruses used in our studies. mCherry-C_14-FW_EF70118836=Tick transcoxally infected with mCherry Hypr, 14 dpi, forward read; mCherry-C_14-RV_EF70118844=Tick transcoxally infected with mCherry Hypr, 14 dpi, reverse read; mCherry-P4-FW_EF70118832=mCherry Hypr passage 4 (revertant) in BHK-21 cells, forward read; mCherry-P4-RV_EF70118833=mCherry Hypr passage 4 (revertant) in BHK-21 cells, reverse read; mCherry-R_14-FW_EF70118847=Tick intrarectally infected with mCherry Hypr, 14 dpi, forward read; mCherry-R_14-RV_EF70118848=Tick intrarectally infected with mCherry Hypr, 14 dpi, reverse read; WT-C_14-FW_EF70118834=Tick transcoxally infected with WT Hypr, 14 dpi, forward read; WT-C_14-RV_EF70118835=Tick transcoxally infected with WT Hypr, 14 dpi, reverse read; WT-R_14-FW_EF70118845=Tick intrarectally infected with WT Hypr, 14 dpi, forward read; WT-R_14-RV_EF70118846=Tick intrarectally infected with WT Hypr, 14 dpi, reverse read.
